# Text Mining Effectively Scores and Ranks the Literature for Improving Chemical-Gene-Disease Curation at the Comparative Toxicogenomics Database

**DOI:** 10.1371/journal.pone.0058201

**Published:** 2013-04-17

**Authors:** Allan Peter Davis, Thomas C. Wiegers, Robin J. Johnson, Jean M. Lay, Kelley Lennon-Hopkins, Cynthia Saraceni-Richards, Daniela Sciaky, Cynthia Grondin Murphy, Carolyn J. Mattingly

**Affiliations:** 1 Department of Biology, North Carolina State University, Raleigh, North Carolina, United States of America; 2 Department of Bioinformatics, The Mount Desert Island Biological Laboratory, Salisbury Cove, Maine, United States of America; Belgian Nuclear Research Centre SCK/CEN, Belgium

## Abstract

The Comparative Toxicogenomics Database (CTD; http://ctdbase.org/) is a public resource that curates interactions between environmental chemicals and gene products, and their relationships to diseases, as a means of understanding the effects of environmental chemicals on human health. CTD provides a triad of core information in the form of chemical-gene, chemical-disease, and gene-disease interactions that are manually curated from scientific articles. To increase the efficiency, productivity, and data coverage of manual curation, we have leveraged text mining to help rank and prioritize the triaged literature. Here, we describe our text-mining process that computes and assigns each article a *document relevancy score* (DRS), wherein a high DRS suggests that an article is more likely to be relevant for curation at CTD. We evaluated our process by first text mining a corpus of 14,904 articles triaged for seven heavy metals (cadmium, cobalt, copper, lead, manganese, mercury, and nickel). Based upon initial analysis, a representative subset corpus of 3,583 articles was then selected from the 14,094 articles and sent to five CTD biocurators for review. The resulting curation of these 3,583 articles was analyzed for a variety of parameters, including article relevancy, novel data content, interaction yield rate, mean average precision, and biological and toxicological interpretability. We show that for all measured parameters, the DRS is an effective indicator for scoring and improving the ranking of literature for the curation of chemical-gene-disease information at CTD. Here, we demonstrate how fully incorporating text mining-based DRS scoring into our curation pipeline enhances manual curation by prioritizing more relevant articles, thereby increasing data content, productivity, and efficiency.

## Introduction

The Comparative Toxicogenomics Database (CTD; http://ctdbase.org) is a public resource that provides information about the interaction of environmental chemicals with gene products, and their effect on human disease [**1]**–**[5**]. This information is garnered from peer-reviewed scientific literature by biocurators who manually curate a triad of core interactions describing chemical-gene, chemical-disease, and gene-disease relationships [**6**].

While manual curation by professional biocurators is recognized as a gold standard of data acquisition for biological knowledge and discovery, it is nonetheless a time-consuming process, and with the encroaching data deluge, it is becoming more challenging to keep up with the growth of published information [**7]**–**[12]**. To make manual curation as efficient and productive as possible, CTD has developed a number of strategies, including the use of a versatile paradigm that multiplexes vocabularies to maximize curation possibilities and minimize data entry requirements [**6**], the development of an efficient web-based Curation Tool accessible by remote biocurators [**6**], the creation and adoption of practical controlled vocabularies to allow data import and integration [**13**], and the initiation of targeted journal curation as an efficient means to increase data currency [**14**]. Finally, we have studied the potential benefits of using text mining to help rank articles and identify curation actors [**15**].

Text mining is becoming an integral step in the curation pipeline for the retrieval and extraction of biological information at curated databases [**16]**–**[18]**. Most notably, WormBase [**19**] has effectively leveraged machine-learning methods to categorize the literature [**20**]; biocurators at WormBase have also successfully used the text-mining application ‘Textpresso’ [**21**] to find and extract a subset of Gene Ontology terms from the full text, increasing curation efficiency by 8- to 15-fold [**22**].

With limited resources and wide-ranging requirements, database groups often develop unique ways to prioritize their literature for manual curation [**23**]. Articles slated for curatorial review at CTD are first triaged from PubMed [**24**] using queries for a particular chemical-of-interest from our priority list [**6**]. These articles are retrieved based upon descending PubMed identification number (PMID), which typically reflects the date of publication; thus, newly published articles generally are at the top of the list awaiting curatorial review, while older papers trend more towards the bottom. This workflow is adequate for chemicals with limited publications, since small corpora can be entirely reviewed and processed by biocurators. For well-studied chemicals, however, the number of potentially relevant articles is often too large for a biocurator to review completely. For example, exposure to heavy metals may influence human health [**25**], and heavy metals toxicity is an important and active area of toxicology research, as evidenced by the more than 33,000 articles with publication dates going as far back as 1926 (http://www.ncbi.nlm.nih.gov/pubmed?term=heavy%20metals%20toxicity). With limited resources, however, CTD simply cannot review such large corpora. Instead, we explored the possibility of using text mining to help make curatorial decisions.

Initially, CTD performed a Phase 1 feasibility study of using text mining by leveraging third party open-source recognition tools (for chemical, gene, and disease entities), associated in-house recognition tools, and ranking algorithms [**15**]. These tools were collectively integrated into a single use application designed solely to assess the potential benefits of using text mining to help rank articles, identify curation actors, and to compare the relative effectiveness of individual tools and configurations. Testing metrics included two standard information-retrieval statistics: mean average precision (MAP), which gauged document ranking effectiveness [**26**], and recall rates, which measured the fraction of relevant instances that were retrieved for gene, chemical, and disease terms. The study involved a test corpus of 1,600 manually curated documents, with results showing that our rules-based algorithm significantly outperformed our previous baseline ordering (73% vs. 63% MAP, respectively), and overall the tools identified 80% of curated actors [**15**].

As a result of the successful Phase 1 feasibility study, we decided to design, develop, and implement the most successful elements from the study in a fully rationalized text-mining pipeline, and integrate it into CTD's regular curation workflow. We refer to this integration effort herein as Phase 2. In conjunction with implementation of Phase 2, large scale, multithreaded batch-oriented processes were developed to address all aspects of text mining at CTD, including to automatically download abstracts from PubMed, text mine and score the abstracts, provide production process status reporting to software engineers, and to provide pre- and post-curation reporting to biocurators. None of the software that was written in conjunction with the Phase 1 feasibility study was used in Phase 2; it was completely redesigned and rewritten for processing efficiency and robustness. Moreover, many of the steps that were manual performed during the Phase 1 feasibility study were fully automated in Phase 2 as part of the move to production.

Here we report our results for Phase 2 wherein we test the effectiveness of this integration effort against a significantly larger dataset (in comparison to the Phase 1 dataset) by extending and employing our text-mining application to process, calculate, and assign a document relevancy score (DRS) to 14,904 articles triaged for seven heavy metals; of the 14,904 articles, 3,582 were then sent to biocurators for review. We demonstrate that for all measured parameters, the DRS is an effective indicator for predicting the relevance and ‘curatability’ of an article for CTD. This scoring helps us prioritize the literature, resulting in improved efficiency and productivity in the manual curation of chemical-gene-disease interactions.

## Materials and Methods

### CTD text mining and curation pipeline

**Figure 1:**
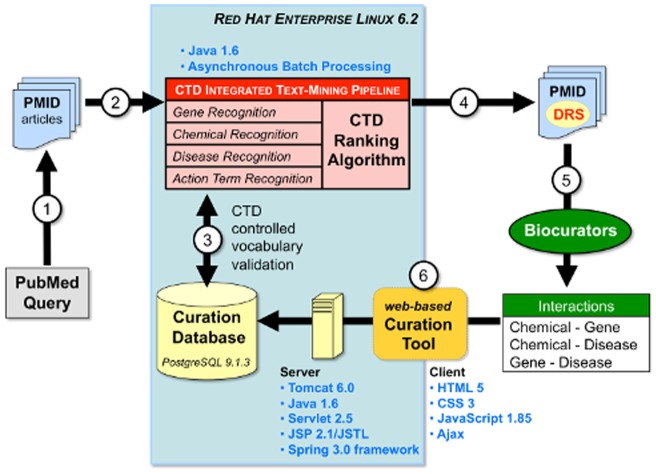
CTD text mining technical overview. (1) A triaged corpus is retrieved for a target chemical-of-interest by querying PubMed. (2) Using the PMID, an article's title and abstract are mined for gene, chemical, disease, and action term recognition in CTD's integrated text-mining pipeline (red box). (3) Each text-mined term is first validated against CTD's controlled vocabularies and ignored if a match is not secured. The CTD text-mining pipeline process is run on a Red Hat Enterprise Linux 6.2 operating system using primarily Java 1.6 within the context of asynchronous batch processes. (4) PMIDs are then assigned a document relevancy score (DRS) by the text-mining tool and (5) sent to biocurators. (6) All interactions are composed and entered in CTD's web-based Curation Tool with the client running HTML 5, CSS3, JavaScript 1.85, and Ajax; a server processes the interactions and stores them in the Curation Database using Tomcat 6.0, Java 1.6, Servlet 2.5, JSP/JSTL, and Spring 3.0 framework.

The CTD text-mining pipeline is divided into several steps (**Figure 1**). First, PubMed (http://www.ncbi.nlm.nih.gov/pubmed/) is queried using CTD-specific strings to triage and retrieve a subset of the literature associated with a target chemical from CTD's ‘Chemical Priority Matrix’ [**6**]. Here, seven independent CTD-specific queries, not involving synonyms, are made for the seven heavy metals cadmium, cobalt, copper, lead, manganese, mercury, and nickel to retrieve 14,904 preliminary articles. The retrieved PMIDs for the articles and the target chemical term are used as input for the text-mining pipeline to produce a ranked list of PMIDs (sorted by DRS) for each text-mined target chemical. Next, the corpus is assigned to biocurators, who use the PMID to access CTD's Curation Tool and initiate curation [**6**]. Finally, post-curation text mining effectiveness-related reports are generated. The reports calculate mean average precision (MAP) [**26**] at the target chemical level, as well as gene, chemical, disease, and action term recall at both the reference and target chemical levels. In addition, details are provided at the reference level, which list text-mined terms, curated terms, and an explanation of how each hit was determined.

### CTD text-mining technical environment and document ranking algorithm

The process for assigning a DRS is accomplished by first extracting each of the PubMed abstracts in XML format (storing the title, abstract text, journal name, and PubMed chemical list). The text of each title and abstract is then mined to identify cited genes/proteins, chemicals, diseases, and action terms. Ultimately, each abstract is scored based upon the CTD rules-based algorithm (**Table 1**). ABNER [**27**] and MetaMap [**28**] are used for gene/protein recognition; in addition, a gene nomenclature normalization process was developed by CTD to normalize gene symbols. Oscar3 [**29]**–**[30]** and MetaMap, as well as each article's PubMed chemical list (if available), are used for chemical recognition. MetaMap is also used for disease recognition. An in-house CTD action term named entity recognition (NER) identifier is also integrated into the text-mining pipeline; the NER identifier stems specific members of CTD's action term vocabulary [**6**] and searches text for instances of their use. The CTD rules-based algorithm, designed and optimized during the aforementioned feasibility study [**15**], assigns points to an abstract based upon specific rules (**Table 1**). The algorithm was developed largely based upon recommendations by biocurators, who conducted an internal analysis of how scientific articles are determined to be curatable during the triage process, and of how highly relevant documents tend to be structured. Internal tools were also developed to identify high throughput-associated abstracts based upon analysis by CTD biocurators. Once the base algorithm was defined, CTD software engineers weighted and optimized the algorithm using multivariate analysis against a test dataset.

**Table 1:** CTD rules-based document ranking algorithm.

Rule	Description	Points
Full text boost	Abstract alludes to additional relevant information in full text, as determined by in-house recognition tools	50
Target chemical in title	Target chemical appears in title; per occurrence	10
Action term co-occurrence	Abstract includes co-occurrence of CTD action terms with genes and chemicals, or genes and diseases, or chemicals and diseases, within a single sentence; per occurrence	8
Target chemical in chemical list	Target chemical appears in MeSH index list	5
Target chemical in first sentence	Target chemical appears in first sentence of abstract; per occurrence	5
Action term occurrence (part 1)	CTD action term appears in abstract, if abstract also contains both genes and chemicals; per occurrence	4
Target chemical in second sentence	Target chemical appears in second sentence of abstract; per occurrence	3
Target chemical in second-to-last sentence	Target chemical appears in second-to-last sentence of abstract; per occurrence	3
Target chemical in last sentence	Target chemical appears in last sentence of abstract; per occurrence	3
Chemical, gene, disease occurrence (part 1)	Chemicals, genes, and diseases appear in abstract, if abstract also contains both genes and chemicals; per occurrence	2
Action term occurrence (part 2)	CTD action term appears in abstract, but abstract does not contain both genes and chemicals; per occurrence	1
Chemical, gene, disease occurrence (part 2)	Chemicals, genes, and diseases appear in abstract, but abstract does not contain both genes and chemicals; per occurrence	1
Priority journal	Journal is *Nature*, *Science*, *Environment Health Perspectives*, *Toxicological Sciences*, *Cell*, or *The Journal of Biological Chemistry*	1

From a software engineering perspective, the pipeline is comprised of multiple asynchronous batch processes that retrieve, score, and report on the PubMed-based abstracts. The key portions of the pipeline are written primarily using Plain Old Java Object (POJOs); each object written in-house, as well as the third party recognition objects described above, are essentially integrated as POJOs, and wrappers were written around the third party objects to standardize input and output. The pipeline runs from within shell-based jobstreams under Linux 6.2; the database management system is Postgres 9.1.3. All text mining is abstract-based; full text is not used. Processing is very fast, typically ranging between 1–2 seconds per abstract and averaged 1.3 seconds per abstract for the heavy metal articles reported in this study.

### Curation of the heavy metal corpus

The subset corpus of 3,583 articles was divided into separate files and assigned to five professional biocurators who have been working with CTD for more than a year and a half. The files contained the PMID, the target metal for which the article had been triaged, and the article title, publication year, and journal name. Biocurators were not provided with the DRS of articles, and PMIDs were listed in descending numerical order to keep biocurators blind to any ranking of the articles. During review, biocurators were asked to perform seven tasks: (1) time themselves using a stopwatch as to how long it took to resolve each assigned article, (2) record if the article should be curated or rejected for CTD, (3) if rejected, provide a short reason why, (4) if curatable, then curate the article for chemical-gene, chemical-disease, and gene-disease interactions, (5) indicate if a curated interaction was garnered from the abstract or full text of the article, (6) record if each curated interaction was studied *in vitro* or *in vivo*, and (7) indicate if each curated interaction was from a high-throughput assay (*e.g*., microarray). Biocurators constructed interactions following CTD's standard curation paradigm using controlled vocabularies, and all data were directly entered in CTD's online Curation Tool [**6**]. The curatorial review of the entire corpus was completed in eight weeks.

## Results

### Project workflow

**Figure 2:**
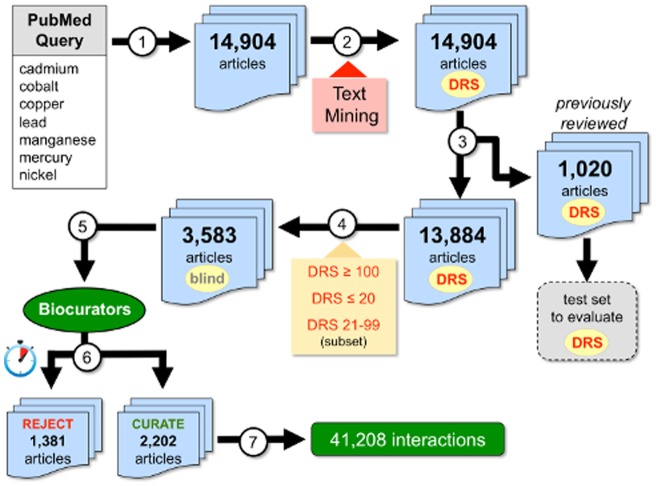
Document workflow. (1) Independent CTD-specific queries were made of PubMed to retrieve 14,904 articles for the seven heavy metals cadmium, cobalt, copper, lead, manganese, mercury, and nickel. (2) These articles were text mined and assigned a document relevancy score (DRS). (3) Of this preliminary corpus, 1,020 articles were found to have been previously reviewed in CTD and were used as a test set to evaluate the DRS and determine suitable cut-offs. (4) Articles with DRS ≥100 (high), DRS ≤20 (low), and a subset with DRS between 21–99 (medium) were combined to provide a final corpus of 3,583 documents which was then (5) sent to five CTD biocurators (who were kept blind to the DRS of each article) for review. (6) Biocurators timed themselves while reviewing all articles and ultimately rejected 1,381 (as non-curatable for CTD) and curated 2,202 of them (7) from whence 41,208 chemical-gene-disease interactions were extracted.

**Figure 3:**
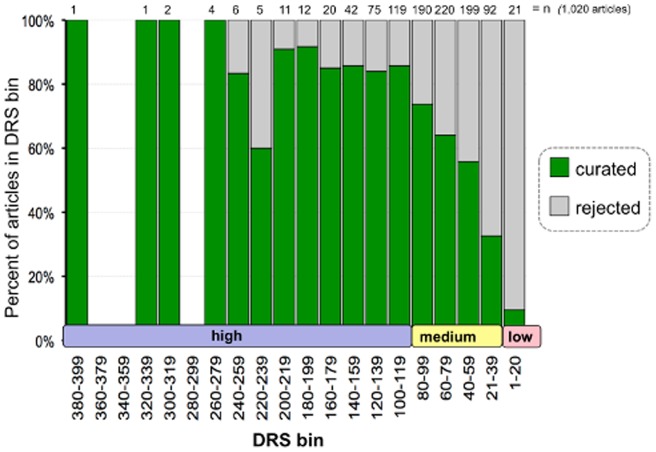
Test set of previously reviewed articles validates assigned DRS. A total of 1,020 articles are distributed by their text-mining assigned DRS (binned in 20-unit increments, *x*-axis) and are indicated as to whether they were found to have been either curated (green) or rejected (gray) by a CTD biocurator (as percent of articles in bin) at a previous time. The number of articles in each DRS bin (n) appears at the top of each column. There were no articles for the bins 280–299, 340–359, or 360–379.

The document flow for this project is outlined in **Figure 2**. Independent CTD-specific queries were made at PubMed to triage and retrieve 14,904 preliminary articles for the seven heavy metals. These articles were then processed by CTD's text-mining algorithm and assigned a DRS, which ranged from 2 to 398 (with higher numbers indicative of presumed increased relevancy). From this preliminary corpus, 1,020 of the articles were discovered to have been previously reviewed by CTD biocurators at an earlier time for different projects. These previously reviewed documents provided a test set to help validate the assigned DRS. We compared the DRS for the 1,020 articles against whether or not the article had been flagged as “curated” or “rejected” in CTD's Curation Tool by previous biocurators (**Figure 3**). Of the 1,020 articles, 86% with a DRS ≥100 were found to be curatable for CTD relevant interactions, while only 10% of articles with a DRS ≤20 could be curated. There is a notably progressive decrease in the percentage of curated articles with a DRS between 21–99, indicating that articles with a DRS ≥100 are likely to be more relevant for curation in CTD while documents with a DRS ≤20 are more likely to be irrelevant. Subsequently, we arbitrarily refer to articles as being in one of three categories based upon their assigned DRS: high (≥100), medium (21–99), or low (1–20).

To test the effectiveness of DRS assignment, we next constructed a subset corpus representative of the 14,904 articles that could be feasibly reviewed by CTD biocurators. From this preliminary corpus, we selected all virgin articles (*i.e.*, articles that had never been examined by CTD biocurators) with a high DRS ≥100 and all virgin articles with a low DRS ≤20 (**Figure 2**, step 4), representing the two ends of the spectrum to allow for the best comparison. To this, we also added all the articles from the mercury subset with a DRS between 21–99 to give a good representation of the medium range; the mercury subset was chosen because it contained a reasonable number of articles that helped to balance the bins in the final assigned corpus. In total, this representative text-mined heavy metal corpus assigned to CTD biocurators for review contained 3,583 articles representing all three DRS categories: 1,981 with a DRS ≥100 (55% corpus), 879 with a DRS between 21–99 (25% corpus), and 723 with a DRS ≤20 (20% corpus). The distribution of articles representing different metals (and their DRS ranges) for this corpus were 28% mercury (DRS: 4–266), 27% copper (DRS: 2–360), 14% manganese (DRS: 4–318), 13% cadmium (DRS: 5–324), 8% cobalt (DRS: 4–284), 6% nickel (DRS: 3–282), and 3% lead (DRS: 8–310).

### Curation metrics

In eight weeks, five CTD biocurators reviewed 3,583 text-mined articles. During review, biocurators (blind to the DRS) decided if an article contained information relevant to CTD, defined as data that describes a chemical-gene, chemical-disease, or gene-disease interaction according to our established paradigm [**6**]. If the document contained relevant information, it was curated following standard CTD procedures into our web-based Curation Tool [**6**]. If the article did not contain any chemical-gene-disease interactions relevant to CTD, it was rejected and flagged as “not curatable” in the Curation Tool. CTD biocurators read and curated the significant points emphasized by the authors in the title and abstract. However, it was sometimes necessary for the biocurator to refer to the full text in order to resolve ambiguities found in the abstract, such as the correct species or gene identity. Once in the full text, the biocurator captured additional essential data not found in the abstract, including relevant information from supplementary tables (*e.g*., microarray tables). Biocurators captured all relevant data for all referenced and resolvable chemicals, genes, and diseases; thus, curation was not restricted solely to the chemical for which the corpus was originally triaged; hence, in this project, interactions were curated for chemicals beyond the seven heavy metals. While entering interactions in the Curation Tool, biocurators designated the source of the interaction as either being derived from the “abstract” or the “full text”.

**Figure 4:**
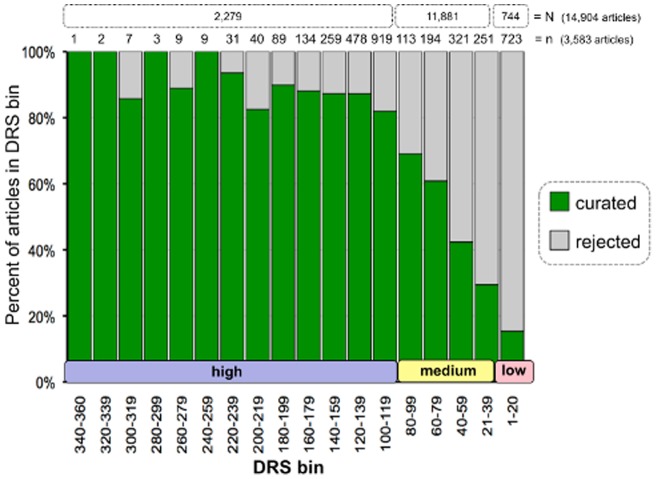
Curation of heavy metal corpus validates assigned DRS. Of the original 14,904 articles (boxes in top row, N), a representative set of 3,583 documents (second row, n) were assigned to CTD biocurators for curatorial review, including all articles (1,981) with a high DRS ≥100, all articles (723) with a low DRS ≤20, and the complete subset of the articles (879) with a medium DRS 21–99 for the heavy metal mercury. (The 1,020 previously reviewed articles were not included in the assigned set.) The articles are distributed by their text-mining assigned DRS (binned in 20-unit increments, *x*-axis) and are indicated as to whether they were either curated (green) or rejected (gray) by a CTD biocurator (as percent of articles in bin). There is a progressive decrease in the percentage of curated articles with DRS <100. In total, 1,685 of the 1,981 articles (85%) with a high DRS ≥100 were curatable, while only 111 of the 723 articles (15%) with a low DRS ≤20 could be curated.

Of the 3,583 examined articles, 2,202 (61%) were curated and 1,381 (39%) were rejected (**Table 2**). We compared the DRS for these articles against their relevancy (*i.e*., “curated” or “rejected”) (**Figure 4**). Similar to the test set of 1,020 documents (**Figure 3**), there was also a dramatic progressive decrease in the percentage of curated articles with a DRS between 21–99 for these 3,583 articles (**Figure 4**). Of the 1,981 articles with a high DRS, 1,685 of them (85%) were curated and only 15% rejected (**Table 2**). Alternatively, of the 723 articles with a low DRS, only 111 of them (15%) were curated and the other 85% rejected, an exact inverse of the high DRS articles. Of the 879 articles that have a medium DRS, 406 (46%) were curated and 473 (54%) were rejected. These metrics reflect the same pattern seen in the test corpus of 1,020 articles (**Figure 3**), and they validate the DRS as a good indicator of an article's relevance for curation at CTD.

**Table 2:** CTD manual curation metrics.

Metric	Total	DRS^a^ ≥100	DRS = 21–99	DRS = 1–20
No. articles reviewed	3,583	1,981	879	723
No. articles curated (% of bin)	2,202 (61%)	1,685 (85%)	406 (46%)	111 (15%)
No. articles rejected (% of bin)	1,381 (39%)	296 (15%)	473 (54%)	612 (85%)
Minutes spent reviewing all articles	38,619	33,471	3,469	1,679
Minutes spent on curated articles (% of bin)	36,342 (94%)	32,660 (98%)	2,900 (84%)	782 (47%)
Minutes spent on rejected articles (% of bin)	2,277 (6%)	811 (2%)	569 (16%)	897 (53%)
Curation rate (± SD)^b^	16.5 (±34.7)	19.4 (±39.0)	7.1 (±7.0)	7.0 (±9.0)
Rejection rate (± SD)^c^	1.6 (±2.0)	2.7 (±3.2)	1.2 (±1.1)	1.5 (±1.5)
No. interactions extracted (% of total)	41,208 (100%)	39,128 (95%)	1,764 (4%)	316 (0.8%)

a DRS  =  document relevancy score bins: high (≥100), medium (21–99), low (1–20).

b Curation rate  =  minutes spent per curated article only. SD  =  standard deviation.

c Rejection rate  =  minutes spent per rejected article only.

Rejected articles consumed only 6% of the biocurators' time (2,277 minutes out of 38,619 total minutes) and averaged 1.6 minutes per rejected article (**Table 2**). The bulk of a biocurator's time (94%) was spent curating articles, with an average curation rate of 16.5 minutes per curated article (**Table 2**). The curation rates correlated with the assigned DRS. Low and medium level DRS papers averaged 7.0–7.1 minutes per article, but documents with a high DRS averaged 19.4 minutes per article (**Table 2**). This progressive rate increase reflects the amount of data extracted from articles. In total, 41,208 interactions were manually curated from this corpus. Of those, 39,128 (95%) were curated exclusively from high DRS articles, and only 4% and a mere 0.8% were extracted from medium and low DRS articles, respectively (**Table 2**).

**Figure 5:**
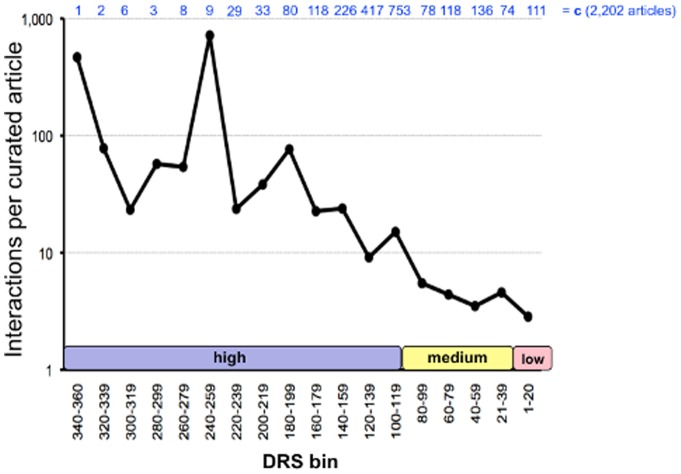
DRS reflects the number of interactions per curated article. Biocurators extracted 41,208 interactions from 2,202 curated articles (top row, c). The average number of interactions per curated article (log-scale, *y*-axis) is distributed by the assigned DRS (binned in 20-unit increments, *x*-axis), with the number of curated articles (c) in each bin indicated at the top. The average number of interactions per curated article increases with the DRS. The aberrant spike in bin 240–259 is due to a single article (amongst a total of nine curated documents in the bin) from whence 5,977 interactions were curated from a microarray experiment.

The number of interactions extracted per curated article also trends with the DRS, demonstrating that documents with a higher DRS have a greater density of curatable information as opposed to articles with a lower DRS (**Figure 5**). Along these same lines, it is interesting to note that it took biocurators almost twice as long to reject a high DRS article compared to a low DRS article, as seen in the rejection rates of 2.7 vs. 1.5 minutes per rejected article, respectively (**Table 2**). We hypothesize that this ∼2-fold difference may be possibly due to the increased density of chemical, gene, and disease actors found in the high DRS documents, requiring a biocurator additional time to sift through all the information before deciding that the article should be rejected.

Of the 2,202 curated articles, CTD biocurators composed interactions for the relevant information from just the abstracts for 1,381 (63%), from both the abstract and full-text for 670 (30%), and from solely the full text for 151 (7%) articles.

### DRS is a better indicator than PMID for ranking the literature

Previous to text mining, PubMed abstracts slated for curatorial review at CTD were ranked solely by descending PMID, which generally reflects the publication date from newest to oldest paper. While this process works fine for small corpora (wherein all the articles will eventually be reviewed by a biocurator), it has major disadvantages for large corpora, since all the articles cannot possibly be reviewed due to limited time and resources; consequently, relevant papers may be missed simply because they have a lower numerical PMID published at an earlier time. Here, with the corpus of 3,583 articles completely vetted by biocurators, we can now retroactively compare the metrics and data content when viewed by either the DRS or PMID ranking methods for a variety of parameters, including (1) article relevance, (2) novel data content, (3) interaction yield rate, and (4) mean average precision (MAP).

For analysis and presentation, the 3,583 articles were first grouped into progressive quartiles (Q1–Q4), each containing 896 documents (except for Q4 which contained 895 articles), based upon either their descending PMID or their descending DRS. Thus, for PMID ranking, articles in Q1 have the highest numerical value (and generally represent the most recently published papers) while articles in Q4 have the lowest numerical PMID value. Similarly, for DRS ranking, documents in Q1 have the highest DRS, which in turn progressively decreases into Q4.

#### (1) Article relevance

**Figure 6:**
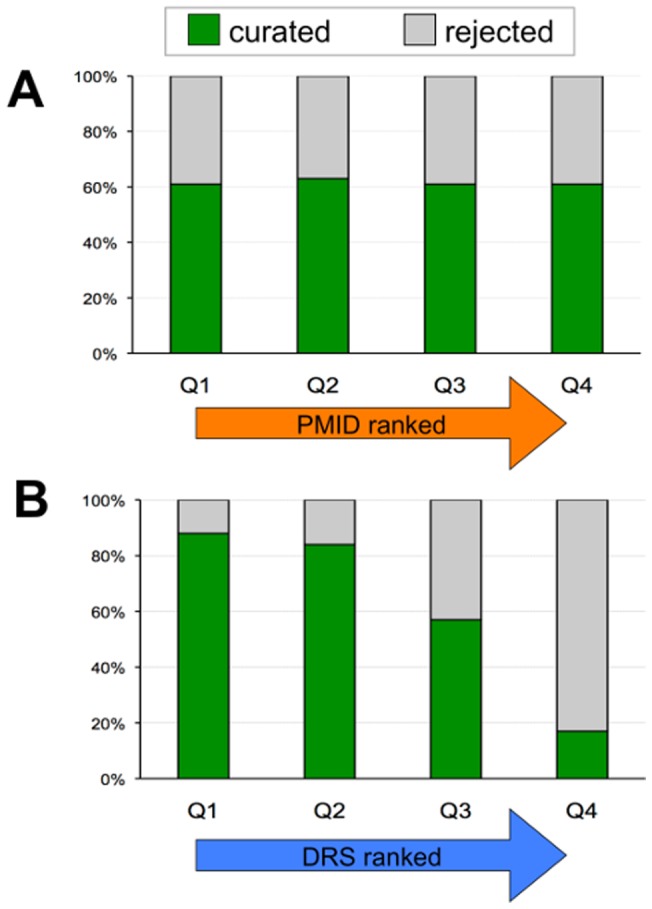
DRS effectively ranks articles for relevance. The 3,583 text-mined articles were ranked via (A) each article's PubMed identification number (PMID) in descending order and via (B) the text-mining assigned DRS, with articles grouped into progressive quartiles (Q1–Q4), each containing 896 documents. The articles were reviewed by CTD biocurators who determined that 2,202 of the articles contained relevant data (curated, green bars) while 1,381 of them did not (rejected, gray bars). The percent of total curated papers vs. rejected papers for each unique quartile are shown.

Of the 3,583 articles reviewed, 2,202 (61%) were curated and 1,381 (39%) rejected (**Table 2**). When an article's relevance (*i.e*., “curated” vs. “rejected”) is viewed by both DRS and PMID ranking, the text-mining tool more effectively scored and ranked the relevant papers via DRS into Q1–Q2, compared to the less informed criteria of PMID, which instead distributed the papers equally across all quartiles (**Figure 6**).

#### (2) Novel data content

**Figure 7:**
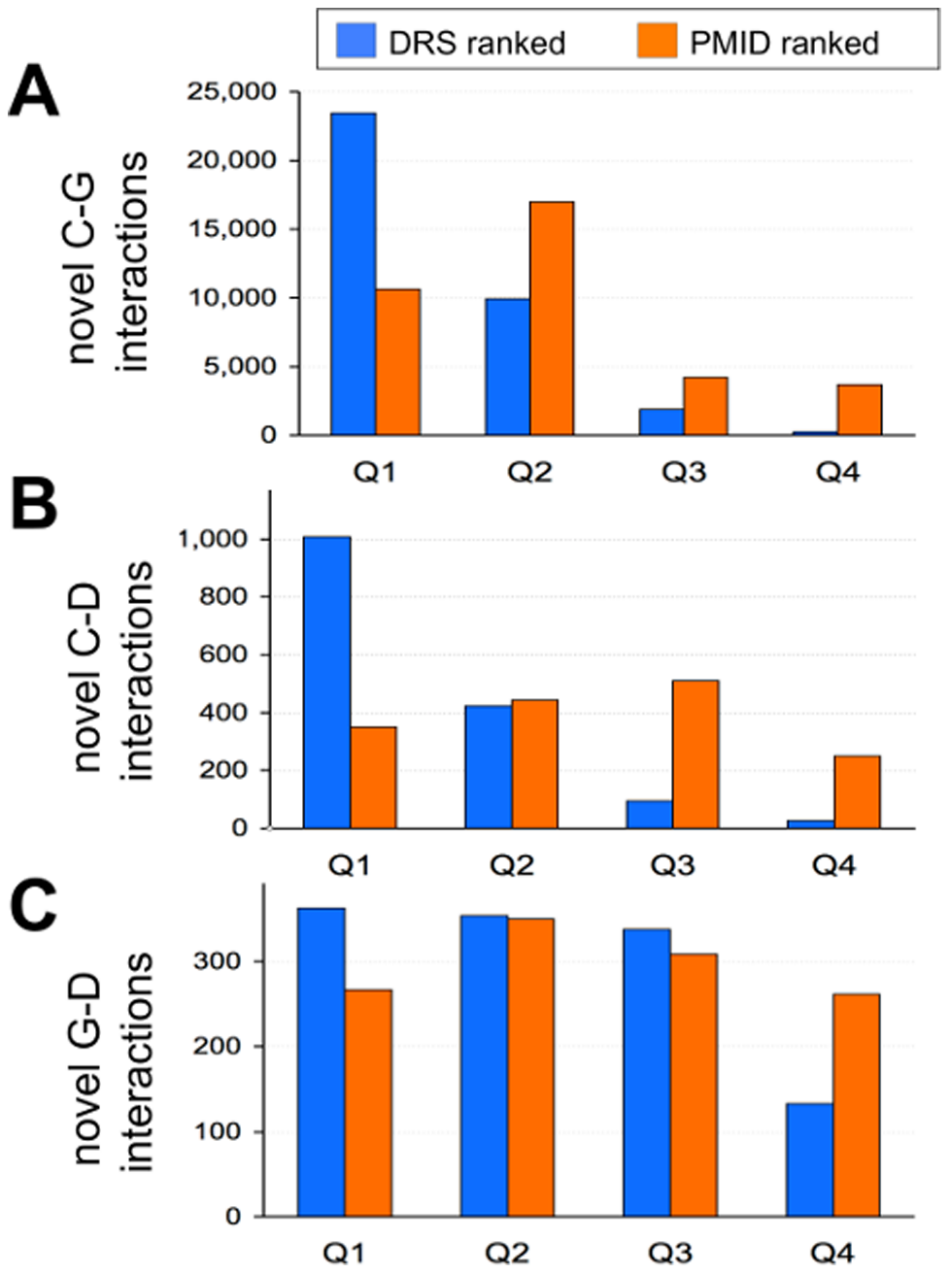
DRS effectively ranks articles for data content. A total of 38,118 novel interactions are distributed into progressive quartiles (Q1–Q4) based upon either DRS ranking (blue) or PMID ranking (orange) for three different types of interactions: (A) 35,385 novel chemical-gene (C–G) interactions, (B) 1,549 novel chemical-disease (C–D) interactions, and (C) 1,184 novel gene-disease (G–D) interactions.

Of the 41,208 total interactions manually curated in this project, 38,118 of them (93%) were novel interactions not yet represented in CTD. The remaining 3,090 interactions (7%) repeated information and provided additional supporting evidence for data that had already been captured from other articles. Biocurators extract three types of information: chemical-gene, chemical-disease, and gene-disease interactions. Since we are interested in discovering new information to be included in CTD, we compared the distribution of the novel content for each type of interaction by both DRS and PMID ranking (**Figure 7**). For all three types of interactions, the DRS more effectively identified and ranked the articles from whence novel interactions were ultimately curated for chemicals, genes, and diseases. Of the 35,385 novel chemical-gene interactions, 23,411 of them (66%) were ranked into Q1 by the DRS method, compared to only 10,617 (30%) by PMID (**Figure 7a**). For chemical-disease interactions, of the 1,549 novel interactions in total, the DRS ranked 1,007 (65%) into Q1 while PMID ranked only 349 (23%) of them (**Figure 7b**). Finally, of the 1,184 novel gene-disease interactions, DRS ranked 31% into Q1 while PMID ranked 22% of them (**Figure 7c**). The somewhat less pronounced differences between quartiles Q1, Q2, and Q3 for novel gene-disease interactions may be due to the more chemical-centric nature of the CTD ranking algorithm itself (**Table 1**). In sum, if curation had been restricted (due to limited resources) to only the first 896 documents (*i.e*., Q1), then ranking the corpus by DRS would have resulted in the collection of 24,780 novel interactions (65% of the possible 38,118 found in the complete corpus), while ranking by PMID would have only generated 11,232 (29%), demonstrating that simply ranking the corpus via text mining resulted in more than a 2-fold increase (65% vs. 29%) in novel data content for this project.

#### (3) Interaction yield rate

**Figure 8:**
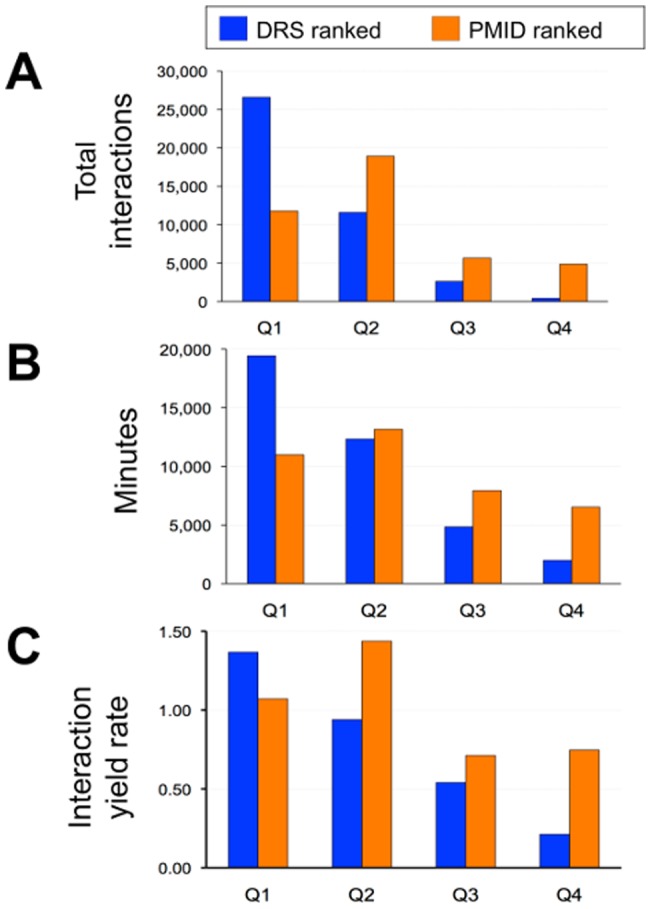
DRS effectively ranks articles for productivity. (A) The number of total interactions (both novel and repeated) for each quartile is divided by (B) the time spent on curating them to produce (C) an averaged interaction yield rate (interactions per minute) for each quartile.

To help evaluate productivity at CTD, we calculate the *interaction yield rate*, defined as the number of interactions curated per unit of time [**14**]. The number of all interactions (*i.e*., novel plus repeated interactions; **Figure 8a**) is divided by the total time spent extracting them (**Figure 8b**) to calculate the average interaction yield rate (**Figure 8c**) for each quartile. Productivity for the first 896 documents in Q1 averaged 1.4 interactions per minute (when ranked by DRS) vs. 1.1 interactions per minute (when ranked by PMID), demonstrating that simply ranking articles by DRS over PMID boosts productivity by 27% for Q1, resulting in more interactions being curated per unit of time.

#### (4) Mean average precision (MAP)

The MAP quantifies the ability of a ranking system to rank relevant documents more highly than non-relevant documents [**26**]. For this study, the MAP for articles ranked by PMID was 62%, but increased to 85% when articles were instead ranked by the DRS method.

Other information-retrieval metrics were also calculated for this study. Gene/protein recall was 71%, chemical recall was 79%, and disease recall was 44% using macro-averaging. Recall scores were calculated by dividing the number of distinct curated gene, chemical, and disease actors identified by the text-mining tools (either by a synonym to the term or by the term itself) by the total number of distinct curated actors. It is important to note that precision (another standard information-retrieval metric) is not appropriate for calculation here. CTD is comprised of curated (rather than cited) genes/proteins, diseases, and chemicals within each abstract. There are many instances where valid, cited actors are not actually involved in curatable interactions, and other instances where curated actors reside only in the full text of the article. Consequently, the complete universe of valid, cited actors specifically resident within each abstract is unknown, preventing the calculation of an accurate precision metric.

### Biological and toxicological interpretability of curated corpus

Although the performance of CTD's text-mining pipeline against the heavy metal corpus is the primary focus of analysis, interpretation of the biological and toxicological aspects of the resulting curation is worthy of note as well. The number of genes (and species from whence they were curated) for each heavy metal was vast, including 3,707 genes from 48 organisms for cadmium, 3,251 genes from 14 organisms for cobalt, 8,004 genes from 47 organisms for copper, 1,078 genes from 16 organisms for lead, 261 genes from 10 organisms for manganese, 462 genes from 45 organisms for mercury, and 1,171 genes from 9 organisms for nickel. The most common species for all seven heavy metals were *Homo sapiens*, *Rattus norvegicus*, and *Mus musculus*, but also prevalent for certain metals were *Danio rerio* (copper), *Macaca fascicularis* (manganese), and *Daphnia magna* (nickel), indicating that a wide range of taxons are used to study heavy metal toxicity and this breadth of research was captured in our pipeline.

CTD biocurators composed 441 unique types of chemical-gene interaction statements, the most prevalent (63%) describing how a heavy metal influenced the mRNA or protein expression of an interacting gene. The remaining 37% chemical-gene statements described heavy metal-gene/protein interactions involving methylation, binding, phosphorylation, activity, localization, secretion, splicing, stability, folding, import, export, cleavage, ubiquitination, chemical sensitivity/resistance, and numerous types of metabolic processing, amongst others. In total, 33 of the possible 55 action terms available in CTD's curation paradigm [**6**] were used to compose interactions for the seven heavy metals, evincing a broad coverage of possible mechanisms of toxicity from the literature.

We next reviewed the gene sets associated with each heavy metal to gauge the biology and putative toxicity derived from this corpus. Gene lists for each metal were compared using CTD's “MyVenn” tool (http://ctdbase.org/tools/myVenn.go) to look for shared and unique genes [**2**]. Sixteen genes were common to all seven heavy metals: CASP3, CAT, GAPDH, HMOX1, IFNG, IGF1, JUN, MAPK1, MAPK3, MT1A, NFE2L2, NFKBIA, NOS2, PTGS2, TGFB1, and TNF. These genes were analyzed using CTD's “Gene Set Enricher” tool (http://ctdbase.org/tools/enricher.go) to find enriched Gene Ontology (GO) biological processes [**2**]. The most statistically significant enriched biological process was *cellular response to chemical stimulus* (GO:0070887), supporting the toxicological relevance of this curated corpus. However, these 16 genes were also enriched for a wide array of other important biological processes, including *gene expression* (GO:0010467; 14 genes), *apoptotic process* (GO:0006915; 13 genes), *regulation of immune system process* (GO:0002682; 11 genes), *cell cycle* (GO:0007049; 9 genes), *neurological system process* (GO:0050877; 8 genes), *response to oxidative stress* (GO:0006979; 6 genes), and the cell signaling pathways *MAPK cascade* (GO:0000165; 6 genes), *Toll-like receptor signaling* (GO:0034130; 4 genes), and *JAK-STAT cascade* (GO:0007259; 4 genes). This diversity suggests there are myriad ways for putative mechanisms of toxicity to be induced by heavy metals.

We also identified genes unique to each of the seven heavy metals curated in this corpus to look for putative metal-specific gene signatures. Of the 3,707 total genes associated with cadmium, 1,708 of them were unique to cadmium when compared against the gene sets for the other six heavy metals for this corpus. The unique gene sets for the other metals were 861 genes (out of 3,251) for cobalt, 4,512 genes (out of 8,004) for copper, 330 genes (out of 1,078) for lead, 30 genes (out of 261) for manganese, 99 genes (out of 462) for mercury, and 240 genes (out of 1,171) for nickel. Many of these refined unique lists were still too large or diverse to deduce any granular metal-specific GO biological processes, though the mercury gene set showed enrichment for *cholinergic synaptic transmission* (GO:0007271; 4 genes), while nickel indicated enrichment for *cytokine-mediated signaling pathways* (GO:0019221; 14 genes), suggesting putative mechanisms of neurotoxicity for the former and immunotoxicity for the latter.

**Figure 9:**
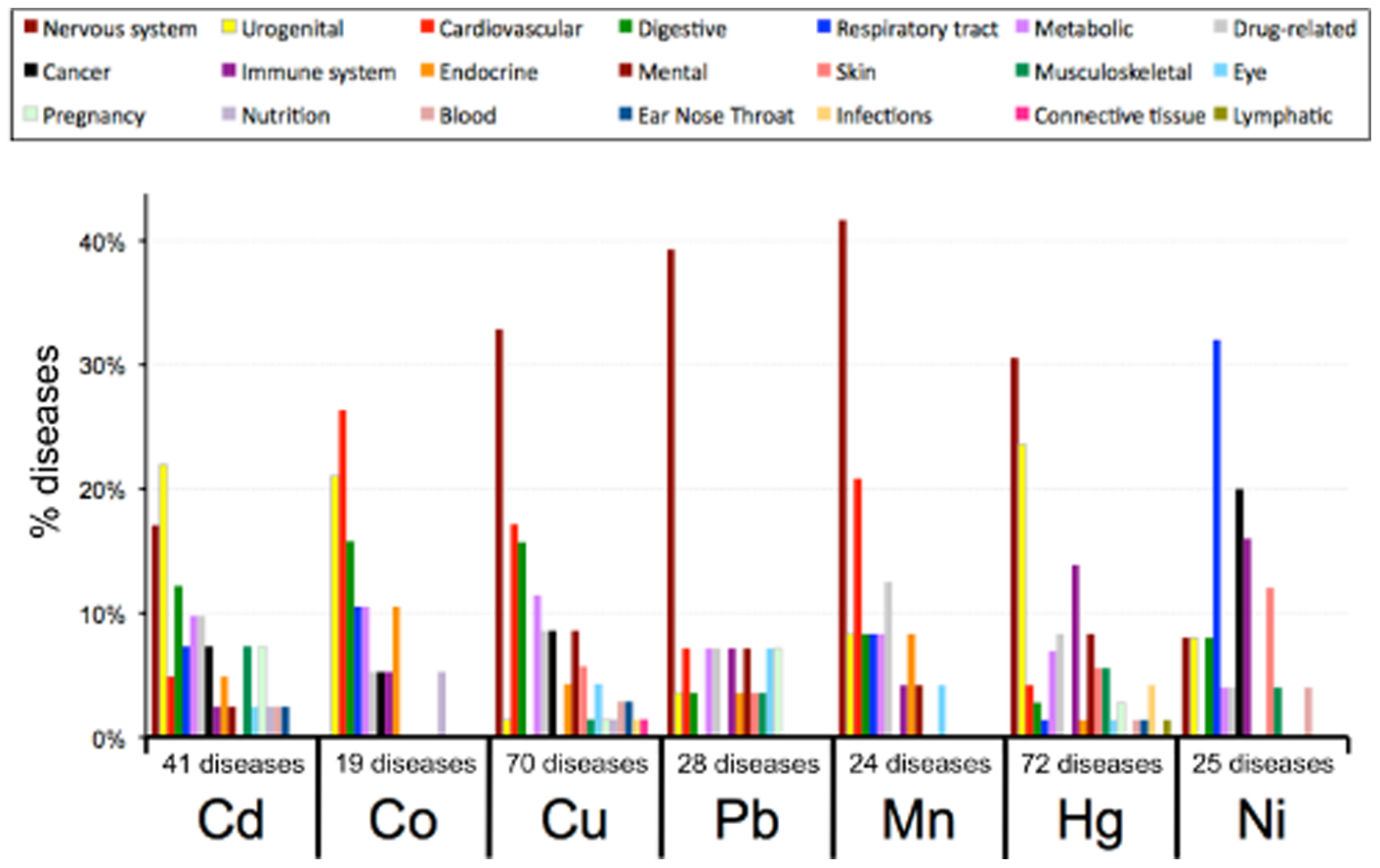
Disease category distribution for the seven heavy metals. The number of diseases curated for each metal is indicated for cadmium (Cd), cobalt (Co), copper (Cu), lead (Pb), manganese (Mn), mercury (Hg), and nickel (Ni). These specific disorders were then mapped and distributed across 21 generic disease categories (legend at top) using CTD's MEDIC-Slim disease mappings [**2**] to look for overrepresented disease classes for each individual heavy metal. For example, of the 70 specific diseases associated with copper (Cu), 23 of them (33%) are nervous system disorders and 12 of them (17%) are cardiovascular disorders.

Lastly, the disorders associated with each of the seven heavy metals included 41 diseases for cadmium, 19 diseases for cobalt, 70 diseases for copper, 28 diseases for lead, 24 diseases for manganese, 72 diseases for mercury, and 25 diseases for nickel. There were no specific diseases common to all seven heavy metals. However, to better visualize this landscape, and to look for shared types of diseases, we mapped these specific diseases to 21 generic disease categories using CTD's MEDIC-Slim disease vocabulary [**2**] to look for common and unique disease classes amongst the metals (**Figure 9**). Copper, lead, manganese, mercury, and cadmium showed a penchant for nervous system diseases, implying a shared toxicity end-point for many heavy metals. Other prevalent disease classes included digestive system disorders (cadmium, cobalt, and copper), urogenital disorders (cadmium, cobalt, and mercury), cardiovascular diseases (cobalt, copper, and manganese), and cancer (nickel, copper, cadmium, and cobalt). Nickel showed the most distinct distribution from the other six metals, with tendencies towards respiratory tract diseases and immune system disorders.

## Discussion

CTD has initiated several processes to improve the efficiency and productivity of manual curation, including the use of a well-defined curation paradigm that leverages multiple vocabularies to maximize curation possibilities and minimize data entry requirements [**6]**, **[13]**, the use of a sophisticated online Curation Tool [**6**], and the introduction of targeted journal curation as an efficient means to increase data currency [**14**]. To this, we have also initiated a text-mining program composed of three phases.

### Phase 1: feasibility study

Phase 1 studied the feasibility of using text mining at CTD by leveraging third party open-source recognition tools, associated in-house recognition tools, and ranking algorithms [**15**]. These tools were collectively integrated into an application specifically designed to assess the benefits of using text mining to help rank articles and identify curation actors (chemicals, genes, and disease terms). The results of this feasibility study using 1,600 manually curated documents demonstrated that text mining could improve article ranking from 63% MAP (baseline) to 73% MAP (algorithm-ranked), and that the tools identified 80% of curated actors, including 74% recall for gene, 94% recall for chemical, and 51% recall for disease [**15**]. The success of the Phase 1 feasibility study encouraged us to proceed with designing, developing, and implementing the most successful elements from the study in a fully automated text-mining pipeline.

### Phase 2: full integration into CTD pipeline

Here, we report our results for Phase 2, wherein we fully implemented a DRS-based text-mining pipeline at CTD to prioritize the literature. With our rules-based algorithm now fully incorporated into the curation workflow, we have tested the effectiveness of this step (post-implementation) against a much larger corpus of 14,904 articles triaged for seven heavy metals. Based upon analysis of a test set, we produced a representative corpus of 3,583 articles spanning three DRS ranges and assayed the validity of the assigned DRS by sending the documents to five CTD biocurators for review. Biocurators performed a series of tasks, ultimately deciding if an article should be “rejected” (and why) or “curated” (and did so). For all metrics, the three DRS bins (*i.e*., high, medium, and low) accurately reflected the article's relevance, the curation rate necessary to review the article, and the density of curatable information contained within the document. Similarly, a head-to-head comparison of DRS vs. PMID ranking showed that DRS is a better indicator of an article's potential ‘curatability’, as evidenced by the first quartile always showing the better results for all parameters when ranked by DRS, with a subsequent and progressive decrease in values for the remaining quartiles. Leveraging DRS resulted in a 37% improvement in ranking over PMID (85% vs. 62% MAP, respectively).

Interestingly, both the test set of 1,020 previously reviewed articles (**Figure 3**) and the assigned corpus of 3,583 articles (**Figure 4**) showed similar patterns of ‘curatability’ reflected by an article's DRS. In the test set, the curatability index was 86% for high DRS, 60% for medium DRS, and 10% for low DRS. Similar metrics were seen for the assigned 3,583 articles, with 85% curatability for high DRS, 46% for medium DRS, and 15% for low DRS. More importantly, however, there was a progressive and consistent decline in curatability in the DRS range from 99 to 21 for both the test set and the assigned corpus, evincing a direct relationship between the DRS and an article's curatability. This linear decline is essential to curation of well-researched target chemicals; if the biocurator is unable to curate an entire target chemical corpus because of its size, it is vital to know that there is a steady and predicable decline in curatability as the biocurator proceeds through the DRS-ranked list of references to avoid spending excessive time on the portion of the corpus that may not be of significant relevance.

With respect to curation metrics, in Phase 2, CTD biocurators spent only 6% of their time rejecting 1,381 articles (39% corpus) and 94% of their time curating 2,202 articles (61% corpus), metrics that parallel our results from the Phase 1 feasibility study [**15**]. However, the overall average curation rate in Phase 2 was 16.5 minutes per curated article, compared to our Phase 1 baseline curation rate of 20.7 minutes per article [**15**]. The reason for this 20% improvement in the curation rate may be due to a variety of factors, including a different corpus size for the timed metrics (3,583 articles in Phase 2 vs. 112 articles in Phase 1) and a different set of biocurators (five biocurators in Phase 2 vs. three in Phase 1). However, we also speculate that a very significant factor is that in Phase 2 we used our online Curation Tool. The original baseline curation rate in 2009 was measured at a time when CTD biocurators exclusively used Excel spreadsheets to compose and store all of their interactions, which was a significantly more manually intensive process. In 2011, however, CTD released a web-based Curation Tool application that obviated the need of spreadsheets and provided numerous time-saving advantages, including built-in quality control measures, real-time validation of entered terms, and a ‘cloning’ feature which allows biocurators to rapidly duplicate interactions and then edit one term to result in a new interaction [**6**].

With respect to text-mining recall metrics, in Phase 2, the tools overall identified 68% of curated actors, including 71% recall for genes, 79% for chemicals, and 44% for diseases, compared to 74%, 94%, and 51% recalls in the Phase 1 feasibility study, respectively [**15**]. Although actor recall was not the primary focus of this study, we believe that these somewhat divergent recall scores are due to significant differences in the corpora. The gross number of gene, chemical, and disease actors curated for the Phase 1 study was 6,664; for Phase 2, the number was much larger: 35,357. Although on the surface it would seem that the larger sample size would yield a more accurate recall measurement, more careful analysis indicates that this may not be the case in this instance. The difficulty in accurately calculating recall at CTD lies in the fact that although text mining is limited exclusively to abstracts, the biocurator often refers to the full text for curation. Moreover, in the case of high-throughput studies (*e.g*., microarray experiments), the vast majority of the curatable data lies in the full text rather than the abstract. In Phase 2, abstract-only curation accounted for 63% of all the curated articles, but only produced 35% of all the curated interactions. Although these statistics are unfortunately not available for Phase 1, we attempted to compare the data that was available for each phase as it potentially relates to recall. Large numbers of interactions in individual papers are typically indicative of high-throughput studies or, at a minimum, instances where the preponderance of data is found in the full text rather than the abstract. For Phase 1, there were no articles with more than 1,000 interactions, and six articles with more than 100 interactions; these six high-density references accounted for 21% of all curated interactions. For Phase 2, however, there were six articles with more than 1,000 interactions, and 34 articles with between 100–1,000 interactions; these combined 40 high-density references accounted for 53% of all curated interactions. The overall average number of interactions per curatable article was 8.4 (SD ±31.1) for Phase 1; for Phase 2, the overall average number of interactions per curatable article was 18.7 (SD ±152.2). Clearly there were significant differences in the corpora. The best case scenario for measuring actual recall is abstract-only (rather than full text) curation, since CTD text mining is limited to abstracts. Although macro-averaging was used in both phases to minimize the distorting effects of full text-based curation on recall calculation, the available statistics associated with the Phase 1 corpus would seem to suggest a greater preponderance of abstract-only curation as a result of its seemingly comparative dearth of full text-based references, and lower average interaction density per curatable article. Consequently, the Phase 1 articles would appear to be somewhat more suitable for recall calculation than the Phase 2 articles. Irrespective of whether this is indeed the case, it is important to stress that recall was extremely effective for purposes of informing the DRS-based ranking algorithm for Phase 2. More time will be spent on this issue during Phase 3 (below), because recall will be of even greater significance when these tools are used to highlight text in abstracts for direct curation by CTD biocurators.

With respect to DRS ranking, the improvement in MAP score from 73% (Phase 1) to 85% (Phase 2) is also probably largely attributable to the difference in the corpora and sample size from the two phases. Nevertheless, a perfect text-mining application would rank every relevant article to the top of the list. Here, even with our successful DRS ranking, we still found relevant articles distributed to the last quartile, as well as articles without curatable data in the first quartile. Upon examination of these outliers, we identified several common reasons for rejection of articles in the first quartile, as well as reasons for curation of articles in the last quartile. The main reason for rejection (representing more than a third of the rejected articles) recorded by the biocurator was due to the lack of an interaction between a chemical and gene, chemical and disease, or gene and disease. In many cases, these articles received a high DRS due to the presence of multiple chemicals or genes in the abstract, but no direct interaction was described. Additional reasons for rejection included descriptions of interactions in species that CTD does not curate (*e.g*., plant, fungus, and bacteria), negative data, and data from review articles. Articles with a low DRS that contained curatable information revealed several elements that were more readily recognized by a biocurator than by the algorithm, including abbreviations, acronyms, and descriptions of gene complexes or general chemical classes. In many cases, the biocurator could resolve these terms directly, or knew to access the full text for more detailed information on the interactions. Modification of the algorithm to reject or lower the scores of papers containing certain species, modification of point values to highlight interactions, and expansion of the library of terms used to reject certain data while recognizing curatable data are all possible strategies that will be examined to improve alignment of DRS with curatability of the articles going forward.

### Phase 3: integration into Curation Tool

Phase 3 will involve integrating text mining directly into CTD's automated Curation Tool [**6**]. Phases 2 and 3 were segregated into individual activities because integrating text mining into our Curation Tool will be a complex process, involving significant modifications to the CTD data model and a complete re-engineering of certain aspects of the Curation Tool. Implementation of a DRS-based pipeline as a standalone project (Phase 2) accommodated a much quicker turnaround largely as a result of its encapsulated nature, and allowed CTD to enjoy the most important benefits of text mining (*i.e*., DRS-based document ranking) sooner than if Phases 2 and 3 had been combined into a single, significantly larger and more complex project.

In addition to ranking documents using DRS, the existing CTD text-mining pipeline identifies gene, chemical, disease, and action terms, and marks up the abstracts with HTML, hyperlinking the genes, diseases, and chemicals back to CTD. Although the recognition data is used as input to inform the DRS ranking algorithm, currently neither the recognition data, nor the HTML mark-up, is used for any other purpose. Phase 3 of the project will integrate these elements into the Curation Tool.

Currently, CTD's Curation Tool is PMID-centric. Biocurators copy the PMIDs from the ranked article list, paste them into the tool, and begin curation. Although this feature will always be available to biocurators, in Phase 3 the tool will become more chemical-centric. In this scenario, biocurators will begin the process by selecting a target chemical-of-interest to curate. The literature corpus for this target chemical will already have been text mined using asynchronous batch processes. Once a target chemical is selected, a list of DRS-ranked PMIDs will be displayed for the biocurator to use. The biocurator will curate directly from within the tool, which will display a marked-up abstract with hyperlinked text-mined chemical, gene, disease, and action terms, providing fully integrated, seamless functionality between the scientific literature and CTD's curation terms in the database. This mark-up will inform, but will not replace, the manual curation process.

Phase 3 will also involve additional analysis and modification of the ranking algorithm with input coming from the outliers described above.

### Comparing CTD text-mining effectiveness with other tools

CTD recently confirmed the relative effectiveness of its text-mining pipeline in conjunction with its participation in organizing BioCreative 2012 Track I [**17**]. CTD was chosen by the BioCreative subcommittee as a source for Track I data because CTD possesses a large and high quality set of curated information of broad interest and relevance to the biomedical research community, as well as significant staff experience with text mining and curation. In September 2011, Track I issued an open invitation to text-mining teams to develop a system to assist biocurators in the selection and prioritization of relevant articles for curation at CTD. Participants were asked, among other things, to rank articles from a relatively small test dataset of 444 articles, as well as use recognition tools to identify cited genes, chemicals, and diseases. Training materials and data sets were provided to the participants so that they would have a complete understanding of CTD curation. A web site providing comprehensive metrics was developed and made available to participants to enable them to test and refine their tools against CTD data in an iterative fashion over a period of months. The participants formed their own teams, sometimes across multiple institutions; a total of seven teams participated, including groups from China, Switzerland, and the United States. The results were impressive: MAP scores were in excess of 70% for all teams, and exceeded 80% for one of the participants [**17**]; top recall scores for gene, disease, and chemical recall were 49%, 65%, and 82%, respectively. However, CTD's tools outperformed all the participating systems overall, and in nearly every individual benchmarking metric, including MAP score [**17**]. One of the participating teams had better disease recall and another had a better action term recall, but CTD placed second in both cases [**17**]. The relative effectiveness of CTD's text-mining pipeline in comparison with other tools is not altogether unexpected when tested against CTD corpora; staff understanding of the CTD domain is obviously extensive, as has been the experimentation with text-mining tool integration (15), and these represent real advantages to CTD. However, the fact that the CTD pipeline fared well when compared to tools developed by teams who, unlike CTD, specialize in text mining, who tailored their applications specifically to CTD curation and corpora, and who used a wide variety of text mining methods (e.g., support vector machines-based, term frequency-inverse document frequency-based, rules-based, etc), was constructive.

### Evaluation of curated content

All of the curated interactions for this corpus have been fully integrated with public CTD and are freely available for researchers to explore. The magnitude and extent of the different types of genes, organisms, interactions, and diseases curated for each heavy metal indicates the broad and encompassing scope to which our curatorial review has covered this knowledge space for the seven heavy metals. Shared, as well as unique, biological processes and toxicological end-points were identified. Importantly, our findings paralleled results from a recent review on metal-induced toxicity [**25**], including the influence of heavy metals on cellular oxidative stress pathways, which was also suggested by our GO enrichment analysis. As well, specific metal-disease relationships described in the review were also covered in our curation for copper (cancer, neurological disorders, diabetes, and cardiovascular disease), cobalt (lung diseases), cadmium (testicular urogenital disorders), and lead (hypertension, neurological disorders, and cognitive impairment). Finally, internal analysis of our data helps corroborate biology with toxicity. The mercury-specific gene set (99 genes) was enriched for a neurological GO process (*cholinergic synaptic transmission*; GO:0007271), which was independently paralleled by this metal also showing a penchant for nervous system diseases (**Figure 9**). Likewise, the nickel-specific gene set (240 genes) was enriched for an immune-related GO process (*cytokine-mediated signaling pathways*; GO:0019221) that was independently supported by immune system disorders being an overrepresented disease class for this metal. Thus, in total, the findings associated with this study support the biological relevance of heavy metal toxicity data and suggest that DRS rankings can successfully inform the process of identifying articles that contribute to an extensive mechanistic understanding of toxicity pathways.

### Summary

With limited resources and the encroachment of larger data landscapes, manually curated databases need to leverage a variety of techniques to maintain and enhance their products. Towards that end, CTD is pioneering strategies to maximize our curatorial resources to increase productivity and efficiency. Here, we have shown how CTD has leveraged text mining to assign a DRS to articles slated for curatorial review as a method of scoring and ranking the triaged literature, with measured improvements in article relevance, novel data content, and increased productivity. It is important to emphasize that not only has CTD constructed an effective DRS scoring-based text-mining program, but as well, we have made DRS scoring fully operational and integrated as part of our regular curation pipeline. This study validates that DRS is effective at ranking the relevant literature for chemical-gene-disease curation at CTD.
